# Highly Porous Para-Aramid Aerogel as a Heterogeneous Catalyst for Selective Hydrogenation of Unsaturated Organic Compounds

**DOI:** 10.3390/polym15153206

**Published:** 2023-07-28

**Authors:** Sergey A. Lermontov, Nikita E. Vlasenko, Nataliya A. Sipyagina, Alena N. Malkova, Inna O. Gozhikova, Alexander E. Baranchikov, Evgeniya I. Knerelman

**Affiliations:** 1 Institute of Physiologically Active Compounds of Federal Research Center of Problems of Chemical Physics and Medicinal Chemistry of the Russian Academy of Sciences, 1 Severnij pr., Chernogolovka 142432, Russia; gmxten@yandex.ru (N.E.V.); sipyagina.nataliya@gmail.com (N.A.S.); amalkova81@gmail.com (A.N.M.); innagozhik@gmail.com (I.O.G.); 2 Kurnakov Institute of General and Inorganic Chemistry of the Russian Academy of Sciences, Leninsky prosp., 31, Moscow 119991, Russia; a.baranchikov@yandex.ru; 3 Federal Research Center of Problems of Chemical Physics and Medicinal Chemistry of the Russian Academy of Sciences, 1 Ac. Semenov avenue, Chernogolovka 142432, Russia; kge@icp.ac.ru

**Keywords:** para-aramid, aerogel, PABI, porous structure, supercritical drying, palladium, catalysts, hydrogenation

## Abstract

A new para-aramid aerogel based on a polymer made by the reaction of terephthaloyl dichloride with 2-(4-aminophenyl)-1H-benzimidazol-5-amine (PABI) is introduced. The aerogel readily bound Pd (+2) ions and was used as a hydrogenation catalyst in some industrially actual reactions. The new material, which did not contain p-phenylenediamine moieties, was prepared in two form factors: bulk samples and spherical pellets of 700–900 μm in diameter. Aerogels were synthesized from 1% or 5% solutions of PABI in N,N-dimethylacetamide via gelation with acetone or isopropanol and had a density of 0.057 or 0.375 g/cm^3^ depending on the concentration of the starting PABI solution. The specific surface area of the obtained samples was 470 or 320 m^2^/g. Spherical pellets containing Pd were prepared from a solution of PdCl_2_ in PABI and were used as heterogeneous catalysts for the gas-phase hydrogenation of unsaturated organic compounds presenting the main types of industrially important substrates: olefins, acetylenes, aromatics, carbonyls, and nitriles. Catalytic hydrogenation of gaseous hexene-1, hexyne-3, cyclohexene, and acrylonitrile C=C bond proceeded with a 99% conversion at ambient pressure, but the catalyst failed to reduce acetone at 150 °C and benzene and ethyl acetate even at 200 °C. The only product of acrylonitrile hydrogenation was propionitrile. The prepared catalysts showed high selectivity, which is important for the chemistry of complex organic compounds.

## 1. Introduction

Aerogels (AGs) are novel materials with a large specific surface area, high porosity, and low density [1,2]. There are two main types of aerogels: inorganic (e.g., SiO_2_ [3], Al_2_O_3_ [4], and carbon [5,6]) and organic (e.g., nanocellulose [7] and phenol-formaldehyde [8]).

In the last decade, many types of organic, namely polymeric, aerogels have been synthesized by applying the sol–gel protocol to linear polymeric materials. Gels are usually prepared by cooling hot solutions of polymers such as polyvinylidene fluoride (PVDF) [9], poly(4-methylpentene-1) [10], polystyrene (PS) [11,12,13], ultra-high molecular weight polyethylene (UHMWPE) [14,15], and polyimides (PI) [16,17,18,19], followed by supercritical drying (SCD) in supercritical CO_2_ (sc-CO_2_).

Aerogels made from linear polymers often have low strength, which limits their use in many practical applications. In addition, they lack thermal and/or chemical stability. One of the approaches to increase the mechanical strength is the use of para-aramid (PPTA, Kevlar DuPont being the most known) as a basis for polymeric AG preparation.

Para-aramids (PPTAs) are one of the strongest materials known to date, so they can be the material of choice for enforced aerogels fabrication. Unfortunately, the preparation and processing of PPTA is difficult. PPTA has a highly oriented chemical structure, and the strong connections between PPTA chains are performed mainly by hydrogen bonding [20]. This fact can be crucial for PPTA AG manufacturing as a loose aerogel structure needs the separation of polymer chains. The main preparation method of PPTA AGs is the use of KOH/DMSO, NH_4_F/DMSO, or H_2_SO_4_/H_3_PO_4_ systems to convert para-aramid fibers into aramid nanofibers (ANF) [21,22,23,24,25,26,27,28], followed by their gelation and supercritical drying (SCD) or freeze-drying procedure. In addition, an industrial-like procedure for PPTA synthesis from terephthaloyl chloride and aromatic diamines in N-methyl pyrrolidone (NMP) in the presence of calcium chloride is also used [29,30]. The PPTA strength could be further increased by making a PPTA-SiO_2_ AG composite [31]. An interesting application of PPTA AG for the preparation of aramid-on-aramid composites is also described in the literature [28,32].

Aerogels are often used as supports in heterogeneous catalysis because they are very promising materials due to their high mesoporosity and specific surface area, which can lead to higher activity and/or selectivity (Pd, Pt, Co, Cu, Fe, and Ru being the most used metal catalysts supported on aerogels) [33,34,35,36,37,38,39,40,41,42], although polymeric supports are rare. Common polymers (polystyrene, polyethylene, polyacrylates, ring-opening metathesis polymers–ROMP gels) as supports are known for a long time [43,44,45,46] and have some advantages over conventional materials: they are lightweight, not brittle in most cases, and can be prepared in different form factors (fiber, textile or monolith). Unfortunately, common polymers share the disadvantages of polymer aerogels, such as low strength and insufficient chemical and thermal stability. For this reason, PPTA can be a material of choice for catalyst polymeric supports, particularly for AG ones.

Papers describing PPTA-based catalysts or materials that can be used as catalysts are not numerous. Two papers devoted to hyperbranched aromatic polyamides (aramids) describe the application of Pd(0) [47] or Pt(0) nanoparticles [48] on hyperbranched polyamides prepared from trimesic acid and p-phenylenediamine. The Pd catalyst demonstrated high activity in the hydrogenation (30 bar H_2_, 80–130 °C) of C-C double and triple bonds and aromatics. Cum et al. [49,50] demonstrated that oligomeric p-aramids containing multiple terminal amino-groups produce active hydrogenation catalysts being treated with a PdCl_2_ solution. The catalyst converted phenylacetylene to styrene and ethylbenzene with a high yield.

To be a catalyst, a material must carry some catalytic centers, usually metal ions or particles. P-aramids are mainly used to adsorb catalytically active Pd or Pt, but other metal ions, such as Ni(+2), Pb(+2), Cu(+2), Fe(+3), and Hg(+2), also can be adsorbed [51,52]. With an aim to increase the concentration of active amino groups in the PPTA macromolecule, a mixed Kevlar-like polymer was prepared from an equimolar mixture of p-phenylenediamine and 2-(4-aminophenyl)-1H-benzimidazol-5-amine, which reacts with terephthaloyl dichloride [30], but no catalysis was performed on this polymer.

PPTA-based materials are usually fabricated by the reaction of terephthaloyl dichloride (TPC) with either p-phenylenediamine or its mixtures with 2-(4-aminophenyl)-1H-benzimidazol-5-amine (PABI) [53]. In this paper, we present a new para-aramid aerogel based on a polymer made by the reaction of terephthaloyl dichloride only with benzimidazol diamine. To the best of our knowledge, neither the textural properties of this material nor its use for the binding of metal ions for catalytic applications in industrially actual reactions is described in the literature. The new material did not contain p-phenylenediamine moieties, which demonstrated low affinity to transition metal ions, but had twice the amount of tertiary amino groups prone to forming complexes with catalytically active metal ions. According to our expectations, the new aerogel material could have the ability to form strong complexes of Pd(+2) with two tertiary nitrogens belonging to different rigid polymer chains, which could affect the gels’ and aerogels’ texture properties and their catalytic activity. In fact, novel aerogels effectively catalyzed industrially important hydrogenation of C–C double or triple bonds leaving aromatics and carbonyl compounds untouched—a feature differing them from industrial and described catalysts.

In addition, a new form of PPTA AG pellet is demonstrated. This form is convenient for catalytic applications and in some other cases, e.g., shock absorbers, heat and noise insulation, and composite materials fabrication.

## 2. Materials and Methods

### 2.1. Materials

Hereafter, the 5% solution of poly-(benzimidazole-terephthalamide) (PABI, η = 520 Pa∙s) in N,N-dimethylacetamide (DMAA), containing 3% *w*/*w* LiCl (Thermotex, Khotkovo, Russia) is denoted as PABI-5, the 1% solution is denoted as PABI-1. LiCl (99%, Acrus, Moscow, Russia), PdCl_2_ (98%, Silversalt, Moscow, Russia), isopropanol (99%, Chimmed, Moscow, Russia), N,N-dimethylacetamide (DMAA, 99%, Acrus, Moscow, Russia), hexene-1 (97%, Acrus, Moscow, Russia), hexyne-3 (99%, Acrus, Moscow, Russia), ethyl acetate (99+%, Acrus, Moscow, Russia), acetone (99+%, Acrus, Moscow, Russia), acrylonitrile (99+%, Acrus, Moscow, Russia), cyclohexene (99+%, Acrus, Moscow, Russia), and benzene (99%, Sigma-Aldrich Chemie GmbH Taufkirchen Germany) were used as received.

### 2.2. Preparation of PABI-Based Samples

#### 2.2.1. Preparation of PABI-1 Solution

An amount of 1 g of PABI-5 was mixed with 4 mL of DMAA and intensively stirred for 1 h to obtain a transparent 1% *w*/*w* solution.

#### 2.2.2. Preparation of Bulk Aerogels

The PABI-1 or PABI-5 solution (2.5 mL) was put into a 5 mL plastic syringe, and then acetone was added (about 2.5 mL). After gel formation, the nozzle cap of the syringe was cut off with a blade and the gel was pushed out with a plunger. Immediately, the gel was completely immersed into 10 mL of acetone for 24 h with periodical gentle agitation in order to remove impurities and unreacted components from lyogels, after which acetone was removed and exchanged for pure acetone 2 times. Then, the aerogel sample was supercritically dried in CO_2_. The samples are denoted hereafter as **AG-1** and **AG-5,** respectively.

#### 2.2.3. Preparation of Aerogel Pellets

The PABI-1 or PABI-5 solution (5 g) was placed into a syringe and dripped through a squashed needle or without a needle (due to PABI-5 high viscosity) into a high glass cylinder filled with isopropanol. The produced pellets were washed with isopropanol once a day for five days, then the samples were supercritically dried in CO_2_. The samples are denoted hereafter as **SAG-1** (spherical aerogel from PABI-1) and **SAG-5**, respectively.

#### 2.2.4. Preparation of Spherical Aerogel Pellets Doped with Pd

An amount of 0.03 g (0.7 mmol) of dry LiCl was dissolved in 40 mL of DMAA, and 0.06 g (0.34 mmol) of PdCl_2_ was then added and stirred for 24 h to obtain a transparent solution. This solution was added to 10 g of PABI-5 solution and stirred for 24 h. The solution obtained (PABI-1 containing Pd) was dripped through a cut needle into a high cylinder with isopropanol. The produced spherical pellets were washed with isopropanol once a day for five days, then the samples were supercritically dried in CO_2_. The sample is denoted hereafter as **SAG-Pd**.

### 2.3. Supercritical Drying

Supercritical drying in CO_2_ was carried out in an installation composed of a high-pressure CO_2_ pump (Teledyne SSI, Supercritical 24, Park College, PA, USA), a 50 mL steel reactor, and a GO Regulator BPR (Spartanburg, SC, USA) backpressure regulator. The aerogel samples were washed with liquid CO_2_ for 1.5 h at 20 °C at a pressure of 15 MPa; then, the temperature in the reactor was elevated to 55 °C, and the samples were washed with supercritical CO_2_ (15 MPa) for 4 h. Next, the pressure in the heated autoclave was gradually (~1 h) decreased to atmospheric pressure; the autoclave was cooled to ambient temperature and opened.

### 2.4. Characterization of Aerogels

The samples were investigated by low-temperature nitrogen adsorption measurements using a static volumetric method with an Autosorb-1 analyzer (Quantachrome Instruments, Boynton Beach, FL, USA). Before measurements, the samples were flushed with a helium flow and degassed at 80 °C in a vacuum for 4 h. The low-degassing temperature was chosen to avoid destruction of the polymeric sample structure. The specific surface area (*S*_sp)_ of the samples was determined according to the Brunauer–Emmett–Teller (BET) equation [54]:

1W×[(P0P)−1]= 1Wmax×C+C−1Wmax×C×(PP0) 

where *p* is the nitrogen pressure in the sample cell, *P*_0_ is the saturated nitrogen vapor pressure at 77 K, *W* is the weight of nitrogen absorbed at a given value of *P*/*P*_0_, *W_m_* is the weight of the adsorbate in the surface monolayer, and *C* is the parameter of the BET equation characterizing the adsorbent–adsorbate interaction. The value of the total pore volume (*V*_p)_ was determined by measuring the amount of nitrogen adsorbed at the value of *P*/*P*_0_ close to 1. The microporosity of the samples was estimated using the t-method [55]. The pore size distribution curves were plotted using the Barrett–Joyner–Halenda (BJH) method [56]. The error in the determination of *S*_sp_ and *V*_p_ values did not exceed 7% and 10%, respectively.

The bulk densities of the samples were calculated by their mass-to-volume ratio.

The microstructure of the samples was studied using a Phenom ProX benchtop scanning electron microscope (SEM) at an accelerating voltage of 10 kV.

EDX mapping measurements were conducted using a Carl Zeiss NVision 40 scanning electron microscope equipped with an Oxford Instruments X-MAX 80 mm^2^ and operating at 1–20 kV acceleration voltage. For the EDX measurements, 20 kV acceleration voltage and an aperture size of 120 μm were used, and the maximum intensity in the EDX spectra exceeded 10^6^ counts.

Thermal analysis was performed on a TGA/DSC/DTA SDT Q-600 analyzer (TA Instruments) upon linear heating to 50 °C (heating rate of 10 °C/min) in a 250 mL/min argon flow.

Elemental analysis was performed using the atomic emission spectroscopy (AES) method with an iCAP-6500 Duo spectrometer (Thermo Scientific, Waltham, MA, USA). Acid decomposition of **SAG-Pd** samples was carried out in the autoclave system with resistance heating ETAS-6 developed in IMT RUS. The decomposition conditions were as follows: sample weight 10–15 mg; a mixture of 0.5 mL HNO_3_ and 0.3 mL HClO_4_; 220 °C (2 h). After completion of the decomposition procedure, the volume of the obtained solution was brought to 10 mL and ICP-AES measurements were performed.

Infrared Fourier spectroscopy (IR) was performed on a Bruker IFS-113V spectrometer in a 4000–350 cm^−1^ region (CaF_2_, thin film) The PABI-1 solution was smeared on a piece of plain glass, dried at 20 °C under atmospheric pressure, washed with isopropanol three times, and dried at 20 °C under atmospheric pressure.

^1^H NMR spectra were obtained using a Bruker DPX-200 spectrometer (approx. 10% solution of organic substance in CDCl_3_) with TMS as a reference standard. Details of product identification by NMR are shown in Supplementary Files (Figures S1–S3).

### 2.5. Hydrogenation of Unsaturated Compounds

The catalyst (V ≈ 1 cm^3^, 50 mg of Pd-containing spherical pellets, **SAG-Pd**) was placed in a glass tube reactor heated with an oil-bath thermostat, and a flow of hydrogen (1.2 L/h) was bubbled through a liquid unsaturated compound (5 mL) at ambient temperature (~22 °C). The mixture of hydrogen and a vapor of the tested compound was passed through a catalytic reactor at a needed temperature (see Figure S4). The reaction product was collected in a cooled trap. The analysis of a product was performed using the NMR method.

## 3. Results

**Figure 1:**
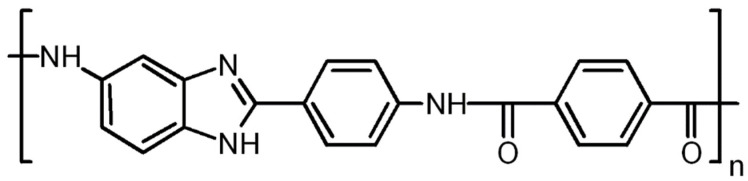
Para-aramid (PABI) structure.

In this paper, we prepared for the first time an aerogel of para-aramid (PABI) containing only 2-(4-aminophenyl)-1H-benzimidazol-5-amine as a diamino component (see Figure 1).

**Figure 2:**
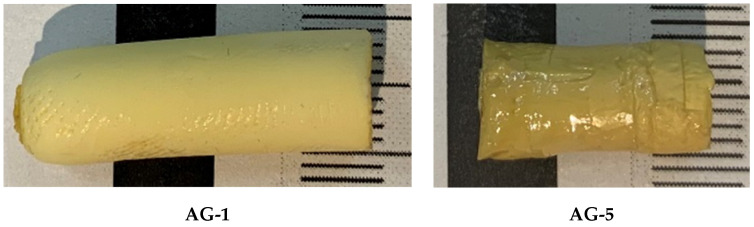
Appearance of aerogels.

Aerogel’s samples were prepared using acetone or isopropanol precipitation of polymer solutions in dimethylacetamide followed by supercritical drying in supercritical CO_2_; the PABI content in the solutions was 1% and 5% (**AG-1** and **AG-5**, respectively). Photos of aerogels are shown in Figure 2.

As can be seen from the photos, the aerogels are monolithic materials. The density of aerogels **AG-1** and **AG-5** was 0.057 and 0.375 g/cm^3^, respectively (see Table 1). IR spectra contained characteristic absorption bands of amide I, amide II-1653, and 1532 cm^−1^, which coincides with the data presented in [30].

**Figure 3:**
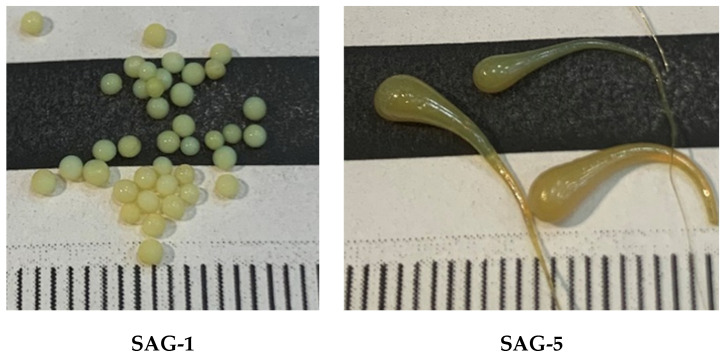
Appearance of aerogels pellets.

Pellets are often used as a convenient form for practical applications, so we decided to produce spherical particles by precipitation of PABI solution dropwise from a syringe into isopropanol, which causes rapid gelation of PABI solutions. It turned out that the 5% PABI solution was too viscous, so the samples prepared were irregularly shaped (Figure 3, **SAG-5**). When 1% PABI solution was used, we obtained particles of a practically spherical shape (see Figure 3, **SAG-1**).

**Figure 4:**
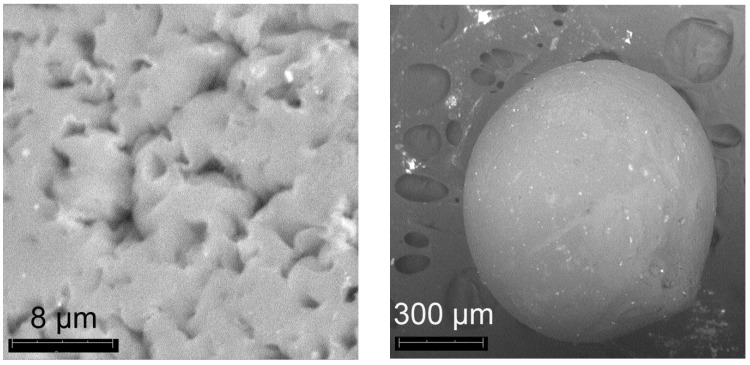
SEM images of spherical aerogel SAG-1 pellets.

Figure 4 shows microphotographs of spherical aerogel **SAG-1** pellets and their porous structure.

**Figure 5:**
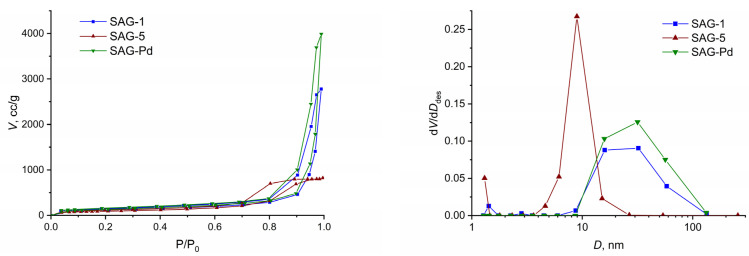
Adsorption–desorption isotherms (**left**) and pore size distribution (**right**).

The textural characteristics of the aerogel pellets were determined using low-temperature nitrogen adsorption. The adsorption–desorption isotherms and pore size distribution are shown in Figure 5. The values of specific surface area and total pore volume for aerogel pellets are shown in Table 1.

All isotherms are of type IV according to the IUPAC classification—which indicates the mesoporous nature of the materials—and are characterized by a narrow H1-type hysteresis loop, typical of materials containing cylindrical open pores [57]. Based on the t-method, it can be concluded that there are practically no micropores in the examined samples. The presence of mesopores is evidenced by the distribution curves of pore volume by size. The pore size values of the **SAG-1** and **SAG-Pd** samples lie in the range of ~6–135 nm (mesopores and small macropores), while the **SAG-5** sample prepared from the more concentrated 5% PABI solution has smaller pores and a narrower distribution in the range of ~4–26 nm (mesopores), with a maximum at ~9 nm.

This correlates with the data in Table 1, according to which, when using a 5% PABI solution, the specific surface area of aerogel pellets is significantly smaller compared to pellets prepared from a 1% solution. The values of the average pore radius and the total pore volume also decrease.

In order to determine the thermal stability of aerogel pellets, we heated samples of aerogel **SAG-1** at 150, 200, and 250 °C in air for one hour/step. The thermal stability of pellets was rather high, though revealed a monotonous decrease in the specific surface area (see Table 2). Even after heating at 200 °C, a drop in *S*_sp_ was within the experimental error. Close results for self-made Kevlar-like aerogel are described in [29], where the material lost approx. 25% of *S*_sp_ after heating at 200 °C for 24 h.

In addition, thermogravimetric analysis was performed. From the thermal analysis data, it follows that both samples, **SAG-1** and **SAG-Pd,** demonstrate virtually similar behavior upon heating in an inert atmosphere. The differences in weight loss for both samples did not exceed 2%. Below 100 °C, the elimination of weakly bound water and residual solvent resulted in a weight loss of ~5%. At temperatures up to ~350 °C, the weight loss is apparently due to the elimination of water and solvents that are bound to aerogel matrix with hydrogen bonds. Heating to 400 °C and higher results in thermal destruction of the para-aramid matrix, which is almost in line with the recently reported data on the thermal stability of para-aramid-based materials [29,58], (Figure S5).

**Figure 6:**
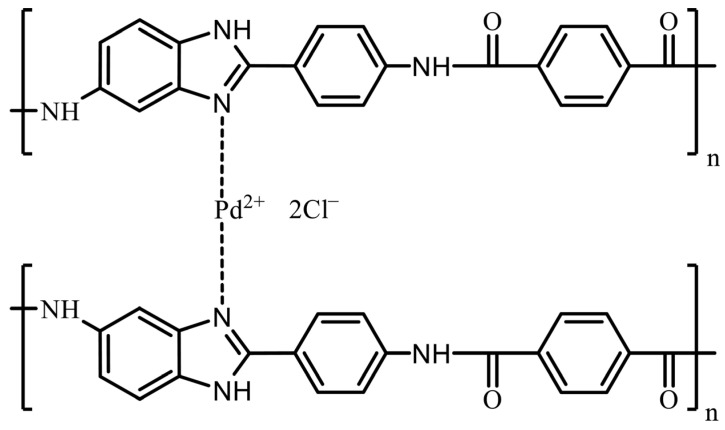
SAG-Pd structure.

**Figure 7:**
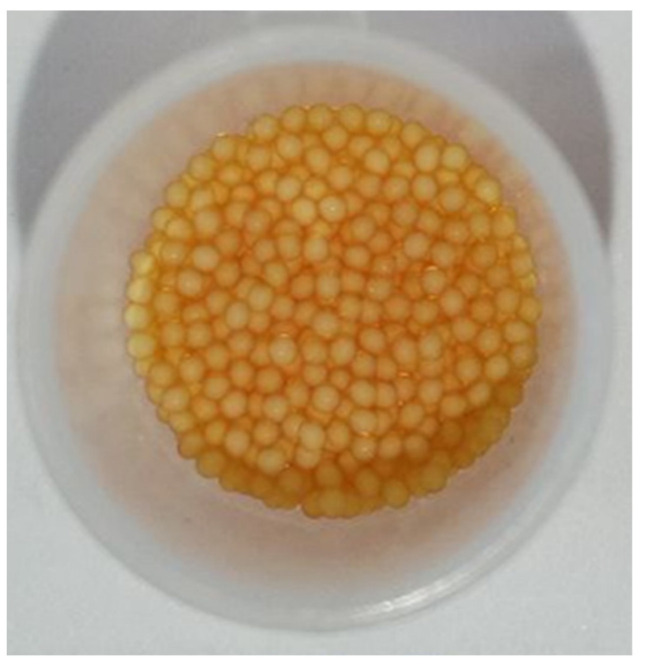
Appearance of **SAG-Pd** aerogel pellets.

Due to the fact that the obtained aerogels had a high concentration of tertiary amino groups in the imidazole cycle, which could be ligands for transition metal ions (no p-phenylenediamine moieties with low affinity to transition metals ions), we introduced palladium (+2) derivatives into spherical samples in order to obtain a convenient form for use in gas-phase catalytic reactions and to determine its catalytic activity. The palladium-containing spherical aerogel **SAG-Pd** pellets were prepared by embedding palladium ions into the PABI-1 solution before gelation in the aerogel preparation step (see Section 2.2.4. and Figure 6 and Figure 7). It is worth emphasizing that the initial concentration of PdCl_2_ implied an approx. 7% Pd load in a catalyst assuming that one Pd ion is bound to one tertiary nitrogen. In fact, according to the AES data, the Pd content was only 0.25 ± 0.01% mass (n = 4; *p* = 0.95). We assume that the larger part of Pd was removed in the gelation and washing steps to leave a strongly bonded Pd between two tertiary amines (Figure 6). Para-aramid gels and aerogels are formed by randomly crossed rigid chains, which differs greatly from a highly oriented hydrogen bonding bulk material. In this structure, the Pd ion “meets” two randomly close-enough nitrogens and forms some kind of bridging between chains. According to the Pd content, it can be supposed that a stable N–Pd–N structure occurs only for ~3% of Pd.

According to EDX measurements, the sample used for the catalytic measurements contained palladium and chlorine at the Pd:Cl atomic ratio of 1:4. Apparently, relatively high chlorine content is due to the synthetic procedure, namely the use of lithium chloride and palladium chloride for the synthesis. The EDX spectra of the samples before and after the catalytic reaction did not significantly differ supporting the high chemical stability of the material. EDX mapping shows a highly homogeneous distribution of palladium in the aerogel matrix; after the catalytic experiment, the homogeneity of palladium distribution was retained with no visible traces of palladium aggregation (see Figures S6 and S7).

**Scheme 1:**
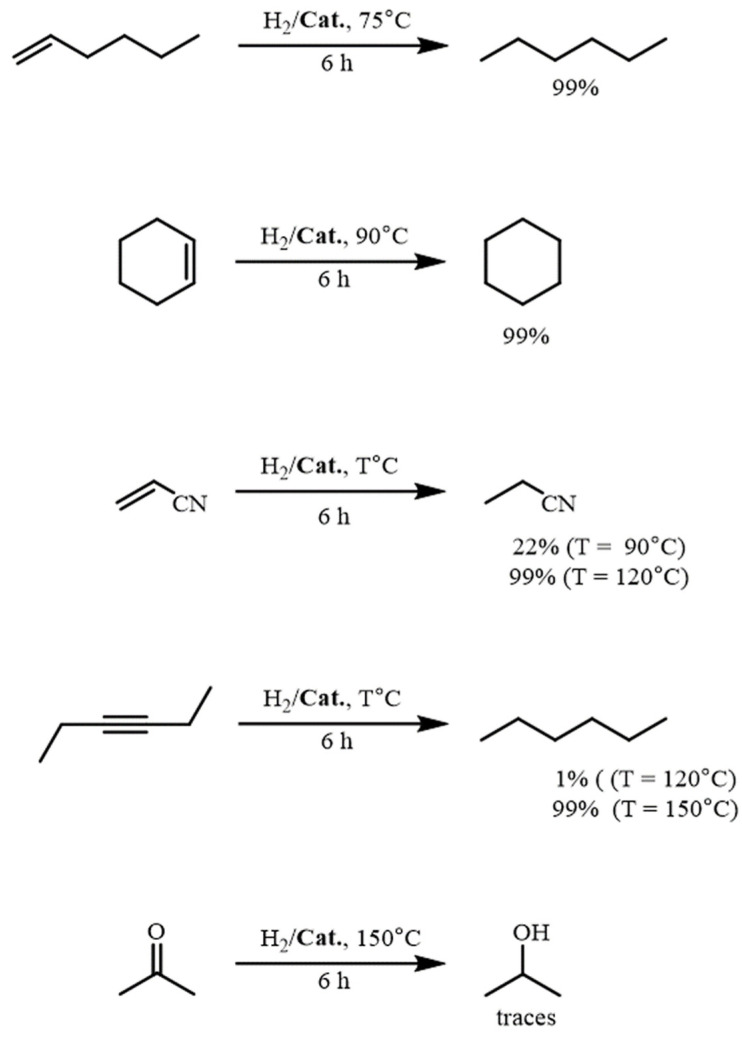
Hydrogenation reactions using a **SAG-Pd** catalyst.

The **SAG-Pd** pellets were not brittle and were strong enough to be treated without special precautions (they could be flattened by strong finger pressure). The aerogel **SAG-Pd** pellets were used as catalysts for gas-phase hydrogenation of organic compounds of various classes, presenting the main types of industrially important reactions (see Scheme 1). All reactions were performed at 1 atm. hydrogen pressure. 

**SAG-Pd** aerogel proved to be an effective catalyst for the hydrogenation of C=C and C≡C bonds in selected organic compounds. In the presence of **SAG-Pd** aerogel, catalytic hydrogenation of gaseous hexene-1, hexyne-3, cyclohexene, and acrylonitrile proceeded with a 99% conversion. The specific surface area of a catalyst sample after hexene hydrogenation (430 m^2^/g) practically did not change compared to the starting catalyst (470 m^2^/g), revealing its chemical stability at least under the reaction conditions. The **SAG-Pd** catalyst failed to reduce acetone or aromatic compounds. It did not demonstrate any activity in the reaction of benzene hydrogenation even at 200 °C, whereas the same reaction in the presence of an industrial catalyst Pd/Al_2_O_3_ (Engelhard) yielded 80% cyclohexane at 100 °C. Ethyl acetate was also not hydrogenated at 200 °C over the **SAG-Pd** catalyst. Acetone hydrogenation using a **SAG-Pd** catalyst resulted only in traces of isopropanol.

The reaction products were identified using ^1^H NMR spectroscopy, some details of the identification procedure are provided in the Supplementary Files section (see Figures S1–S3). The functional reproducibility of the catalyst upon reaction conditions is proved by the fact that after hexyne-3 hydrogenation at 120 °C (1% conversion), the catalyst was further used for hydrogenation of hexyne-3 at 150 °C and provided a 99% conversion to n-hexane. In addition, the retaining of a high conversion value during 6 h of hydrogenation reveals the stability of the catalyst at least under reaction conditions.

It is worth mentioning that Engelhard’s 0.5% Pd/Al_2_O_3_ was taken as a reference highly active catalytic material. Our metal-containing polymeric aerogels were not so active compared to Engelhard’s, but they showed high selectivity in olefins, acetylenes, aromatics, carbonyls, and nitriles hydrogenation. Moreover, the content of Pd in the **SAG-Pd** catalyst was 0.25% compared to 0.5% in Engelhard’s sample. 

## 4. Conclusions

We have found that the **SAG-Pd** catalyst with a low palladium content (0.25%) can be successfully used in fine organic synthesis, e.g., for selective reduction of the C-C double bond in the presence of a triple bond, which is a serious problem in organic synthesis. Additionally, aromatics, nitriles, and carbonyls will not be affected during olefins’ and acetylenes’ reduction. This feature is in some contrast with previously reported results [47], where aromatics, acetylenes, and olefins were successfully hydrogenated over Pd supported on superbranched para-aramid at a hydrogen pressure of 30 atm. Several important patterns concerning the novel aerogel-based catalyst should be emphasized:

- Terminal C=C bond (hexene-1) is more active compared with the 1,2-disubstituted one (cyclohexene);
- Electron-withdrawing group (CN) decreases the C=C bond hydrogenation speed, leaving a CN group untouched;
- The C≡C group (hexyne-3) is much less active in comparison with the C=C group;
- The catalyst retains its activity together with its shape at least up to 150 °C in a hydrogen/organic vapor atmosphere.
- The catalyst reveals a very sharp increase in activity with an increase in reaction temperature from the reduction of hexyne-3. The conversion of a triple bond turned from 1% to 99% within a narrow temperature interval of only 30 °C. The explanation may lie in the reaction mechanism. In the first step, hexyne-3 is converted to hexene-3, which is then quickly reduced to n-hexane. A higher reaction ability of alkenes over alkynes in hydrogenation reactions is known from a general course of organic chemistry [59]. In addition, we found traces of the olefinic bond (probably hexene-3) in the ^1^H NMR spectrum of the hexyne-3 reduction at 120 °C. Therefore, the speed-limiting step of hexyne-3 hydrogenation is the conversion of C≡C → C=C with a fast step of C=C → C-C.

The proposed form factor non-brittle pellets can be convenient for liquid-phase reactions of complex organic compounds as they will be easily removed from reaction mixtures and regenerated. Such reactions are in progress now.

## Figures and Tables

**Table 1:** Textural characteristics of aerogels: specific surface area (*S*_sp_, m^2^/g), bulk density (*ρ*, g/cm^3^), and total pore volume (*V_p_*, cm^3^/g).

Sample	*ρ*, g/cm^3^	*S*_sp_, m^2^/g	*V_p_*, cm^3^/g
**AG-1**	0.057		
**AG-5**	0.375		
**SAG-1**		410	4.3
**SAG-Pd**		470/430 *	6.2
**SAG-5**		320	1.3

* after hexene hydrogenation at 75 °C during 6 h.

**Table 2:** Thermal stability of aerogels **SAG-1** and **SAG-Pd** pellets.

T, °C	20 °C	150 °C	200 °C SAG-1/SAG-Pd	250 °C
*S*_sp_, m^2^/g	410	403	390/430	250

## Data Availability

Not applicable.

## Supplementary Materials

The following supporting information can be downloaded at: https://www.mdpi.com/article/10.3390/polym15153206/s1, Figure S1. ^1^H NMR spectrum of initial hexene-1; Figure S2. ^1^H NMR spectrum of the reaction product after hexyne-3 hydrogenation at 120 °C.; Figure S3. ^1^H NMR spectrum of n-hexane—a hydrogenation product of hexene-1; Figure S4. Schematic hydrogenation reactor; Figure S5. The thermal analysis of SAG-1 and SAG-Pd samples; Figure S6. EDX mapping spectrum of SAG-Pd before hydrogenation reaction; Figure S7. EDX mapping of the aerogel samples (a) before and (b) after the catalytic experiment.
