# Supplementary material for: Highly Porous Para-Aramid Aerogel as a Heterogeneous Catalyst for Selective Hydrogenation of Unsaturated Organic Compounds
Source: Polymers (Basel). 2023 Jul 28;15(15):3206. doi: 10.3390/polym15153206 (PMC10421250; doi:10.3390/polym15153206)
Supplement: Supplementary file 1 [file polymers-15-03206-s001.zip › polymers-2505351-supplementary.pdf]

Supplementary Materials

Highly Porous Para-Aramid Aerogel as a Heterogeneous Catalyst for Selective Hydrogenation of Unsaturated Organic Compounds

Sergey A. Lermontov [1,*], Nikita E. Vlasenko [1], Nataliya A. Sipyagina [1], Alena N. Malkova [1], Inna O. Gozhikova [1], Alexander E. Baranchikov [2] and Evgeniya I. Knerelman [3]

1 Institute of Physiologically Active Compounds of Federal Research Center of Problems of Chemical Physics and Medicinal Chemistry of the Russian Academy of Sciences, 1 Severnij pr., Chernogolovka 142432, Russia; gmxten@yandex.ru (N.E.V.); sipyagina.nataliya@gmail.com (N.A.S.); amalkova81@gmail.com (A.N.M.); innagozhik@gmail.com (I.O.G.)

2 Kurnakov Institute of General and Inorganic Chemistry of the Russian Academy of Sciences, Leninsky prosp., 31, Moscow 119991, Russia; a.baranchikov@yandex.ru

3 Federal Research Center of Problems of Chemical Physics and Medicinal Chemistry of the Russian
Academy of Sciences, 1 Ac. Semenov avenue, Chernogolovka 142432, Russia; kge@icp.ac.ru

* Correspondence: lermontov52@yandex.ru

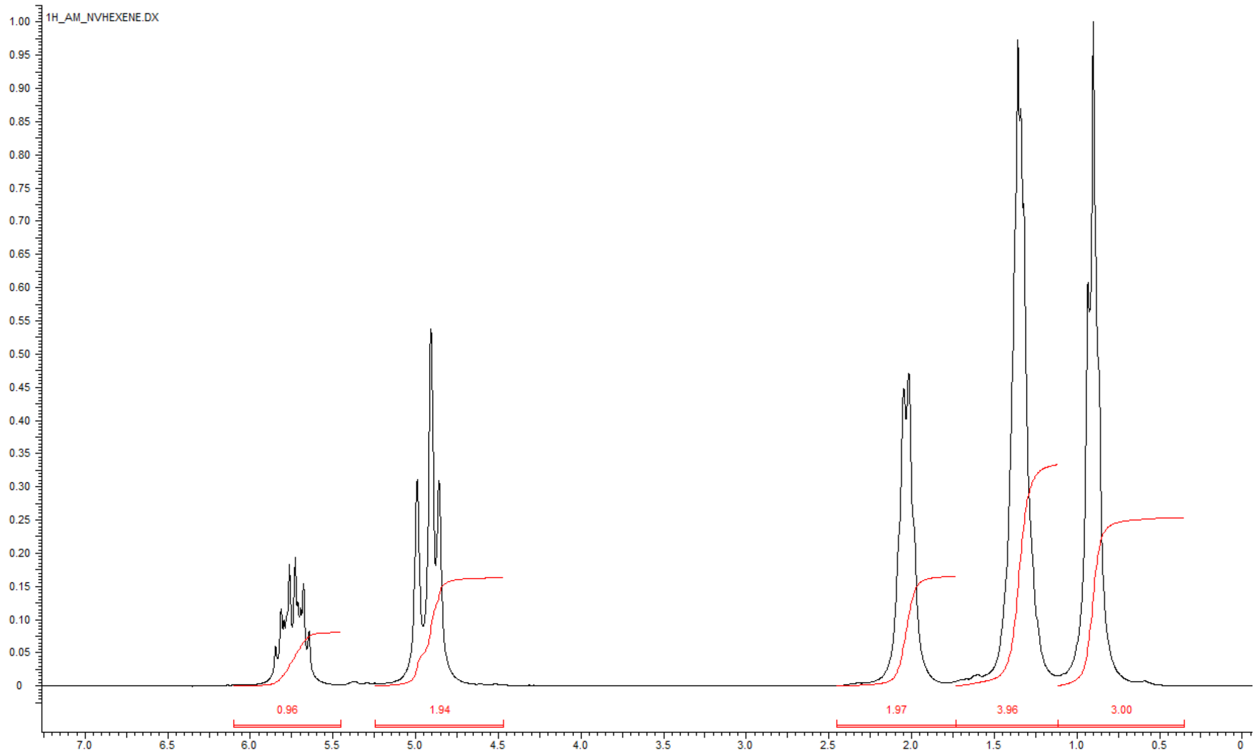

Figure S1. ^{1}H NMR spectrum of initial hexene-1.

Two groups of signals (4.7 - 5.8 ppm, 2:1 integral intensity) refer to CH_2=CH protons - a complex $ABCX_2$ spin system. Three groups (0.7 - 2.2 ppm, 3:4:2 integral intensity) refer to CH_3, (CH_2-CH_2) and CH_2-Csp2 protons respectively.

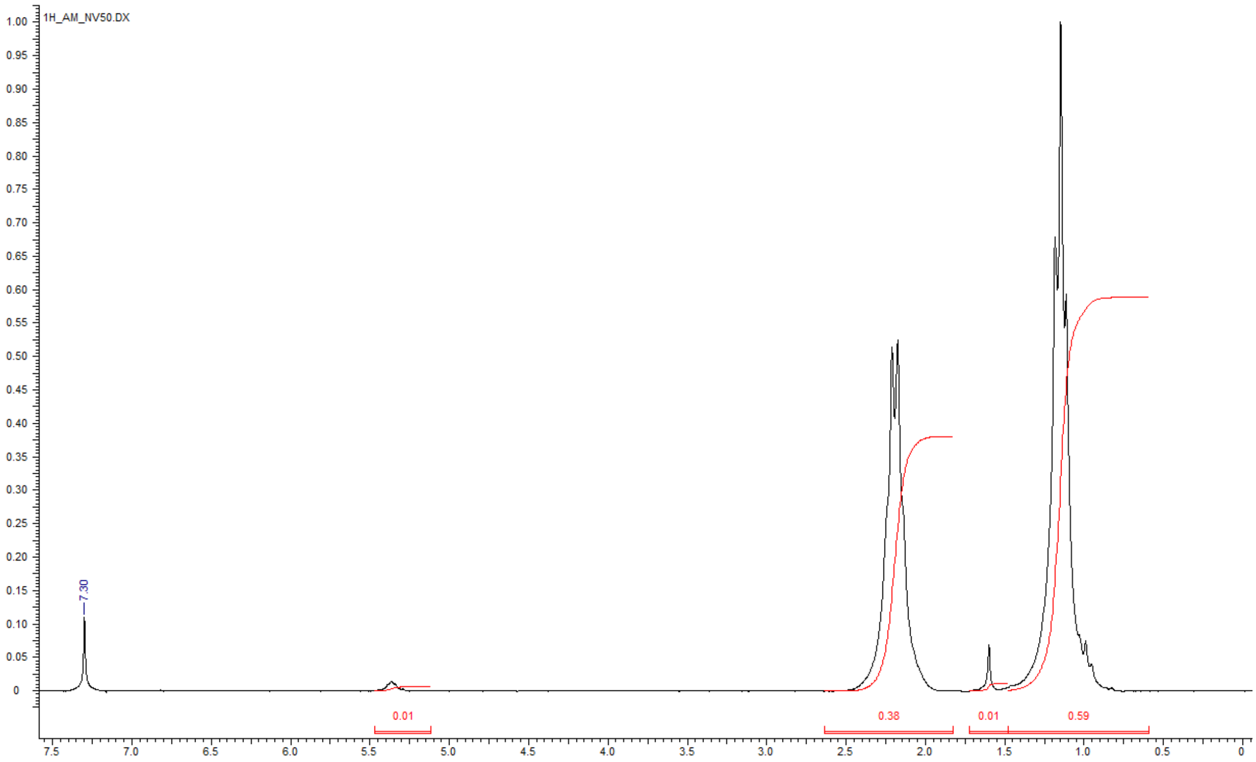

Figure S2. ^{1}H NMR spectrum of the reaction product after hexyne-3 hydrogenation at 120 C.

Two main signals (1.1 and 2.2 ppm, 3:2) refer to CH_3 and CH_2 protons in the initial hexyne-3. A small signal at 5.3 ppm best suits the Alk-CH=CH-Alk protons in hexene-3. Small signals at 1.5 and ~ 0.8 ppm best suit n-hexane protons (see **S4** below).

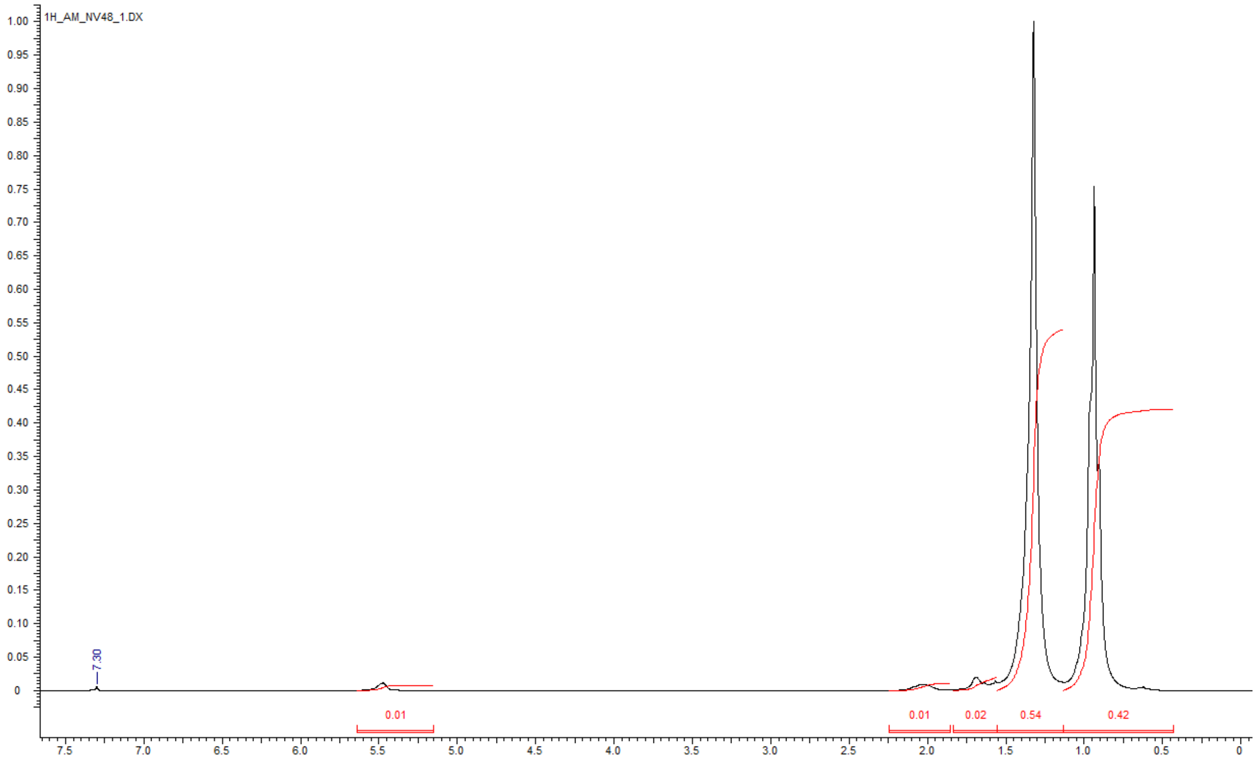

Figure S3. ^{1}H NMR spectrum of n-hexane - a hydrogenation product of hexene -1. In case of hexyne-3 the product was the same

The spectrum consists of two signals (0.8 and 1.4 ppm, 3:4 or 6:8, $2CH_3$ and $4CH_2$ protons). As seen, the spectrum dramatically differs from starting compounds by chemical shifts and signals' form. The same about cyclohexene and acrylonitrile - olefinic protons disappear, cyclohexane singlet signal or propionitrile ethyl group protons appear. All spectra are easy to unequivocally identify the products and distinguish them from starting materials even at partial conversion of substrates.

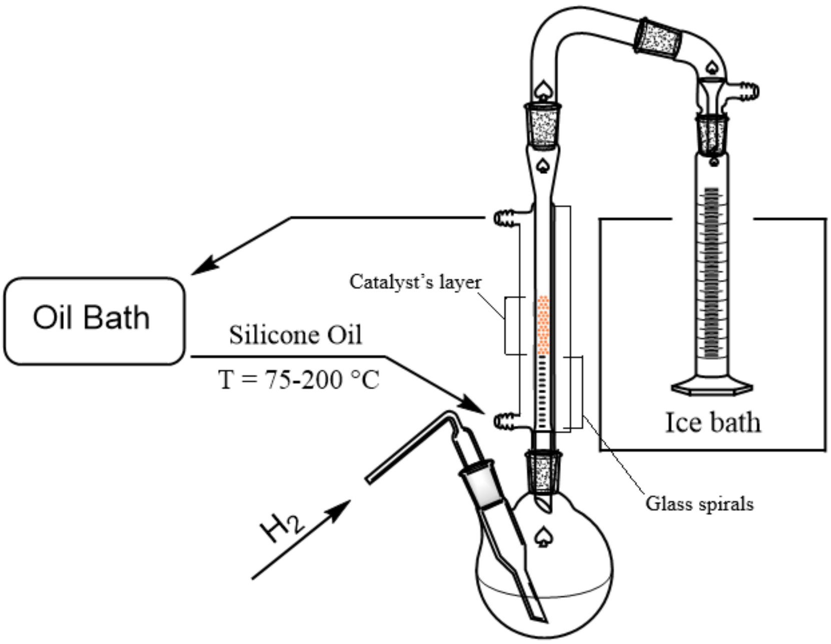

Figure S4. Schematic hydrogenation reactor.

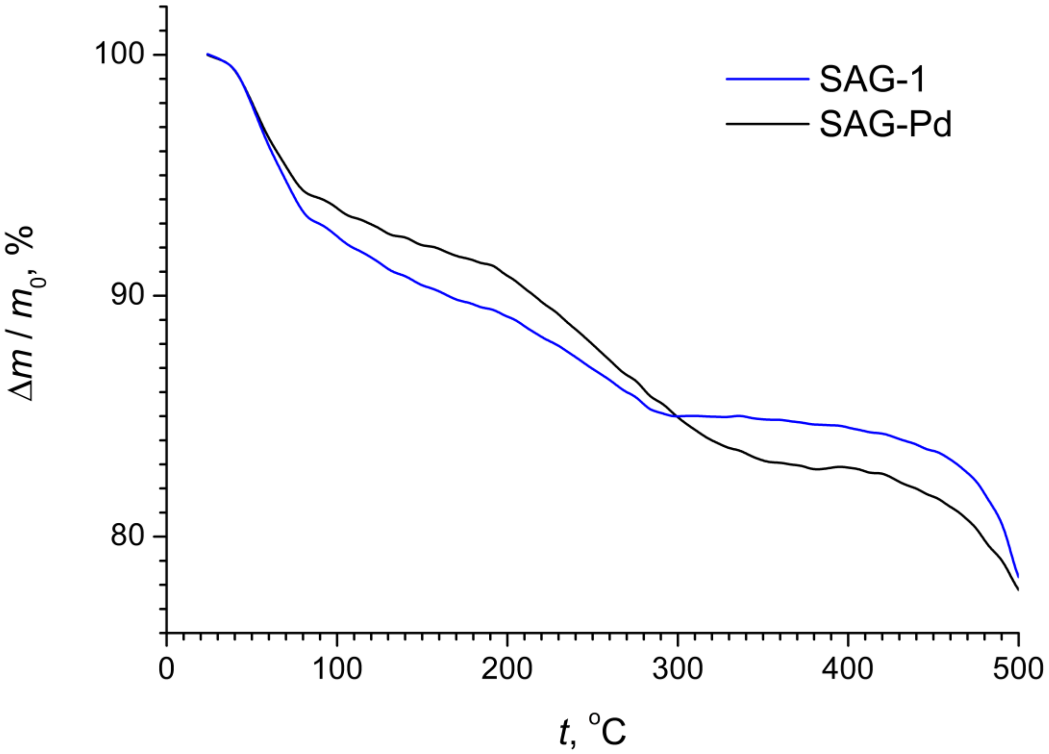

Figure S5. The thermal analysis of **SAG-1** and **SAG-Pd** samples.

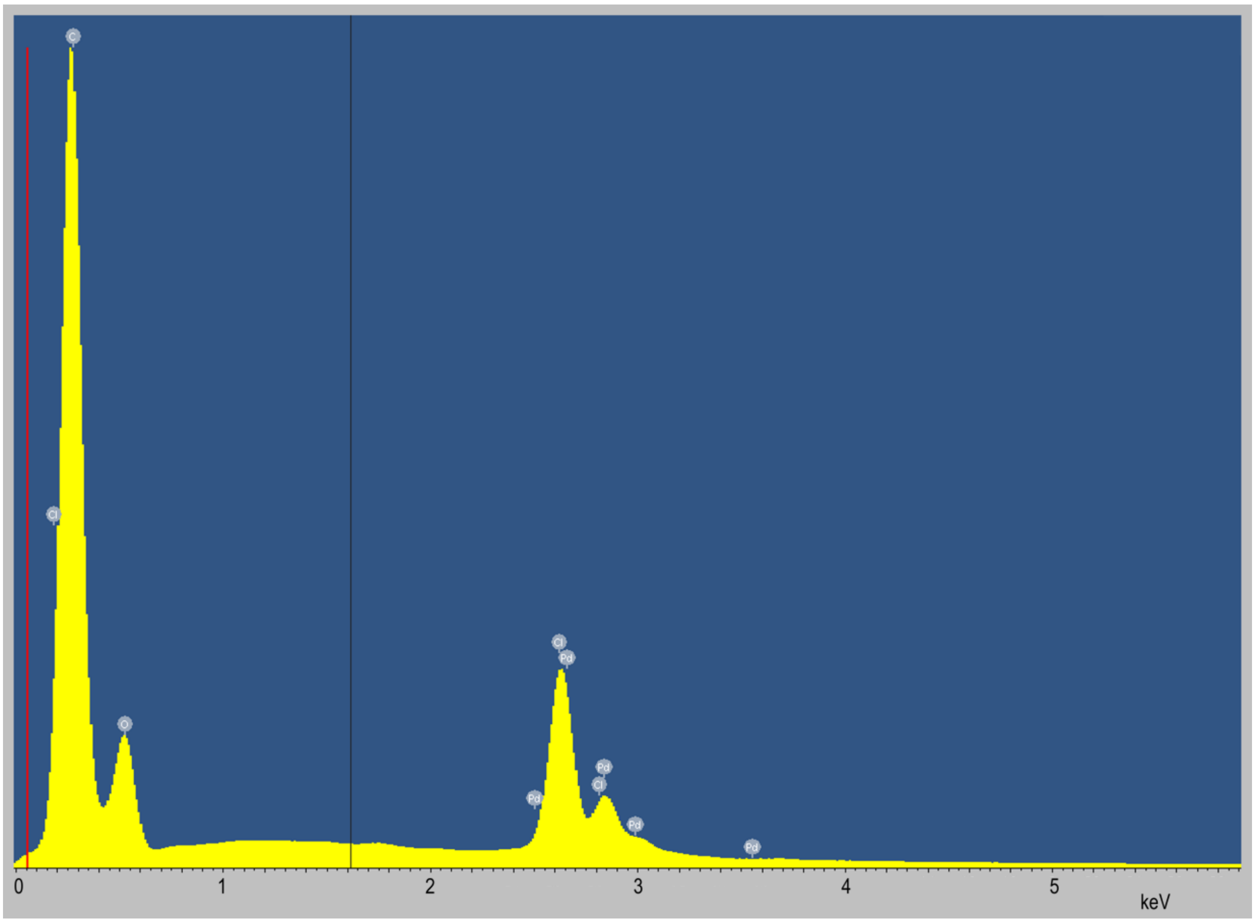

Figure S6. EDX mapping spectrum of **SAG-Pd** before hydrogenation reaction.

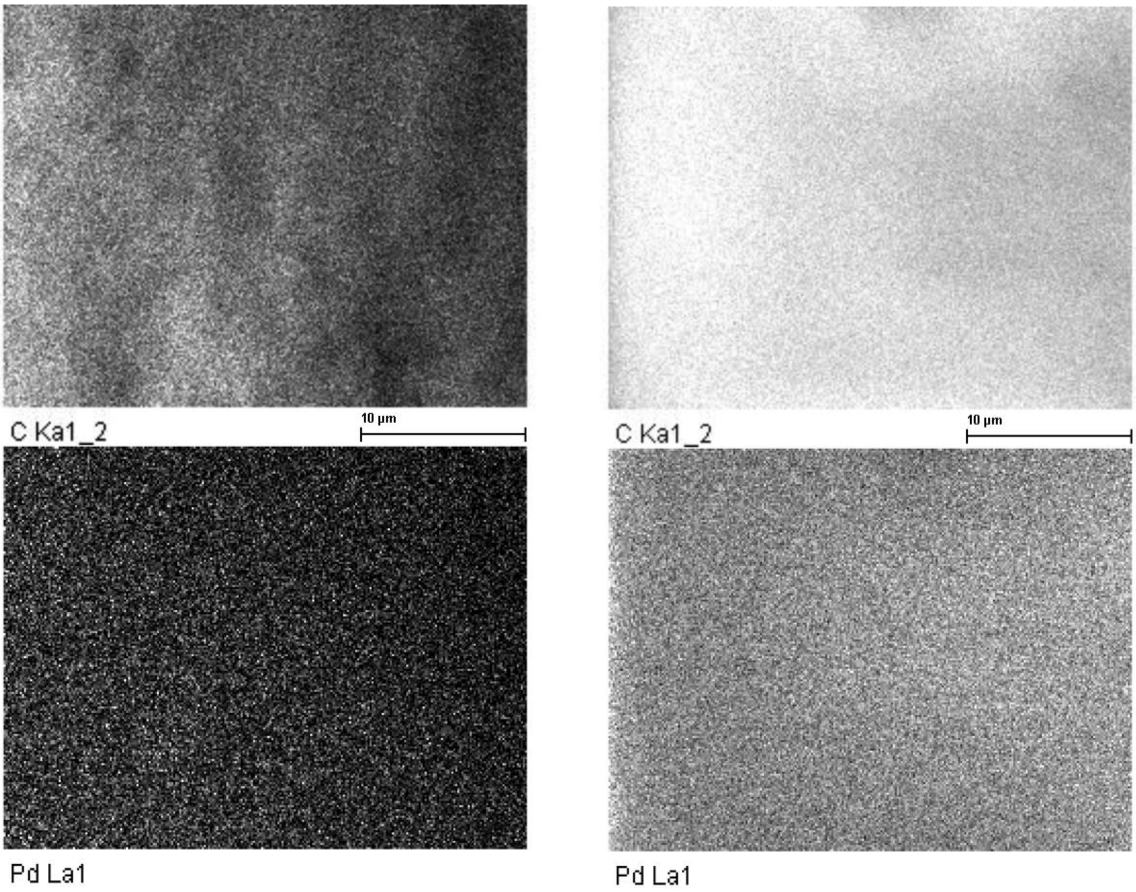

a **b**

Figure S7. EDX mapping of the aerogel samples (**a**) before and (**b**) after the catalytic experiment.
